# MRI based volumetric measurements of vestibular schwannomas in patients with neurofibromatosis type 2: comparison of three different software tools

**DOI:** 10.1038/s41598-020-68489-y

**Published:** 2020-07-14

**Authors:** Philipp Kollmann, Victor-Felix Mautner, Johannes Koeppen, Ralph Wenzel, Jan M. Friedman, Johannes Salamon, Said Farschtschi

**Affiliations:** 1 Department of Neurology, University Medical Center Hamburg-Eppendorf, Martinistr. 52, 20246 Hamburg, Germany; 2 Department of Neurosurgery, University Medical Center Hamburg-Eppendorf, Hamburg, Germany; 3 Radiological Practice Altona, Hamburg, Germany; 4 Department of Medical Genetics, University of British Columbia, Vancouver, Canada; 5 Department of Diagnostic and Interventional Radiology and Nuclear Medicine, University Medical Center Hamburg-Eppendorf, Hamburg, Germany

**Keywords:** Brain imaging, CNS cancer

## Abstract

Neurofibromatosis type 2 is a neurogenetic disorder with an incidence of about 1:33.000. Hallmarks are bilateral benign vestibular schwannomas, which can lead to deafness or brainstem compression. Volumetric tumor measurements are essential to assess the efficacy of new therapies. We present a statistical and methodical comparison of three volumetric image analysis tools. We performed volumetric measurements on phantoms with predefined volumes (0.1 to 8.0 ml) and tumors seen on 32 head MRI scans from eight NF2 patients with BrainLab, ITK-Snap, or OsiriX. The software was compared with regard to accuracy and reproducibility of the measurements and time required for analysis. The mean volume estimated by all three software programs differed significantly from the true volume of the phantoms, but OsiriX and BrainLab gave estimates that were not significantly different from each other. For the actual tumors, the estimated volumes with all three software tools showed a low coefficient of variability, but the mean volume estimates differed among the tools. OsiriX showed the shortest analysis time. Volumetric assessment of MRI images is associated to an intrinsic risk of miscalculation. For precise volumes it is mandatory to use the same volumetric tools for all measurements.

## Introduction

Neurofibromatosis type 2 (NF2) is a rare neurogenetic tumor predisposition disorder with an estimated incidence of about 1:33.000 live births^1^. Hallmarks of NF2 are bilateral vestibular schwannomas (VS), peripheral schwannomas, cranial or spinal meningiomas and ependymomas^2^. VS are benign in nature but can cause severe neurological deficits and deafness as a result of local tumor growth^3^. In 2009 bevacizumab—given off-label for compassionate use—showed tumor reduction and hearing improvement in patients with NF2^4^. The efficacy of this drug on NF2 associated VS has subsequently been confirmed in other studies^5–9^.

Tumor growth rate and treatment response are crucial parameters for risk stratification and therapy monitoring of patients with NF2. 3D image analysis is significantly superior to conventional planar measurements for radiographic monitoring of VS growth (or regression)^10–12^. Numerous software solutions have been developed for tumor segmentation and volumetric analysis^13–16^, but none has emerged as the clinical standard for monitoring tumor growth in NF2 patients.

We here present a comparison of three different volumetric software solutions for measuring VS on cranial MRIs.

## Methods

### IRB

This study and the experimental protocol were authorized by the ethics committee of the University Medical Center Hamburg Eppendorf. All participants gave their informed consent. All procedures performed were in full accordance with the Declaration of Helsinki.

### Phantoms

We used a commercially available make-up storage box with identical compartments (side length 2 cm) to create the larger phantoms. Each compartment was filled with contrast agent (Gadenobate dimeglumine 0.5 M (MultiHance) in a dilution of 1:20 with NaCl) ranging from 1 to 8 ml, using a precision pipette (Rainin Pipet-Lite XLS 0.1–10 ml for 5 ml and 8 ml and Rainin Pipet XLS 100 µl–1 ml for 1 ml to 3 ml). We also produced a set of microphantoms ranging from 0.1 to 0.7 ml (Supplementary Table S1) using micro reaction vessels filled with contrast agent.

### Patients

Eight patients with a clinical and molecular diagnosis of NF2 were included in this study (three females, five males) with a median mean age of 41 years (range 29–67 years) (Table 1). All patients exhibited bilateral vestibular schwannoma; one of the patients had undergone previous surgery. For this study, a total of 32 MRI scans were available for volumetric evaluation. The selected VS of each patient was measured in all consecutive scans respectively. The 8 patients were selected to obtain a range of different tumor sizes from 0.15 to 11 ml (mean 4,5 ml). Follow-up scans were performed about every three months, and the median total follow-up time included in the study was 8.5 months (range 7–26 months) per patient. All patients were on long-term medication with bevacizumab for their vestibular schwannomas (2.5 mg/kg to 7.5 mg/kg 2 to 3-weekly). Three patients (#4, #5 and #6) discontinued medication during follow-up for four, five and three months respectively due to limiting side-effects (proteinuria and abnormal estrous cycle). Treatment duration ranged from 10 to 288 months at last MRI scan with a median duration of 61.5 months.

**Table 1:** Patient characteristics.

Patient number	1	2	3	4	5	6	7	8
Age (years)	67	34	48	32	34	53	54	29
Sex	Male	Female	Male	Male	Female	Male	Male	Female
Bevacizumab treatment at start of study	Yes	Yes	Yes	Yes	Yes	Yes	Yes	Yes
Treatment duration (weeks) at end of study	52	41	66	67	57	288	112	10
Treatment dosage	5 mg/kg 3-weekly	5 mg/kg 3-weekly	3.5 mg/kg 3-weekly	5 mg/kg 2-for 10 weeks; subsequently 2.5 mg/kg 2-weekly	7.5 mg/kg 2-weekly	5 mg/kg 3-for 276 weeks; subsequently 2.5 mg/kg 3-weekly	5 mg/kg 2-weekly for 32 weeks; subsequently 2.5 mg/kg 2-weekly	2.5 mg/kg 2-weekly
Treatment discontinuation	No	No	No	Yes	Yes	Yes	No	No
Follow up time (months)	8	9	7	8	8	26	12	15

### MRI

All scans were conducted at the Radiological Practice Altona in Hamburg, Germany, using a Siemens Magnetom Skyra (3.0 T). For this study, we used T1-weighted high-resolution contrast enhanced acquisition with 192 slices with a slice-thickness of 1 mm without intersectional gaps.

### Software

All MRI scans were evaluated volumetrically three times with each software tool in order to determine intra-rater variability. A total of 43 scans (five phantoms, six microphantoms and eight patients with four scans each) were measured with three different software solutions for volumetric assessment of MRI-based cranial imaging. In total, 387 manual segmentation and semi-automated volumetric measurements were part of the investigation. Analysis time was recorded to evaluate usability of the software (patient #1 to #5).

The three software programs chosen for comparison are:

(1) OsiriX Lite (v.7.0.3.) 32-bit used on an iMac (3,2 GHz Intel Core i5, main memory: 8 GB, 1867 MHz DDR3; graphics: AMD Radeon R9 M390 2048 MB). OsiriX is an macOS-based image processing software for viewing and analyzing DICOM images and a fully functional PACS workstation, including an extensive range of measurement tools for volumetric assessment. For this post-hoc analysis for research purposes only we used the free-of-charge lite version. In this version, which is not approved for clinical diagnostics, the number of image analysis tools is limited but the volumetric analysis is fully functional and has the same processing time as the clinically accredited version. The clinically-accredited version is available through the company’s website (https://www.osirix-viewer.com/)

(2) ITK-Snap (v.2.4.0) 32-bit used on an iMac (3.2 GHz Intel Core i5, main memory: 8 GB, 1867 MHz DDR3; graphics: AMD Radeon R9 M390 2048 MB). ITK-Snap is a freely available open source DICOM-software tool for viewing and segmentation of MRI scans, working on all conventional operating systems^17^.

(3) BrainLab Iplan (v.4.5, BrainLab, Feldkirchen, Germany) is a specialized and medically approved Windows-based (64bit Windows 10) neuronavigational software running on a fully equipped neurosurgical stereotactic workstation (HP 640; 2,4 Ghz, Intel Xeon 12-core; 32 GB RAM; graphics: Quadro M400).

### Statistics

Statistical analyses of volume estimates for phantoms and of estimated tumor volumes in NF2 patients were carried out with R version 3.6.0 (2019–04-26). Comparisons of the actual volumes of the phantoms to the volumes measured with each of the software programs was performed using t-tests, and comparison of estimates obtained by the software programs to each other was done by analysis of variance or, if the data were not normally distributed, the Kruskal–Wallis test. Comparisons of volumes of patient tumors measured with different software programs was performed using repeated measure analysis of variance. Linear regression was used to assess the relationship of phantom volume to coefficient of variation.

## Results

### Phantoms

**Figure 1:**
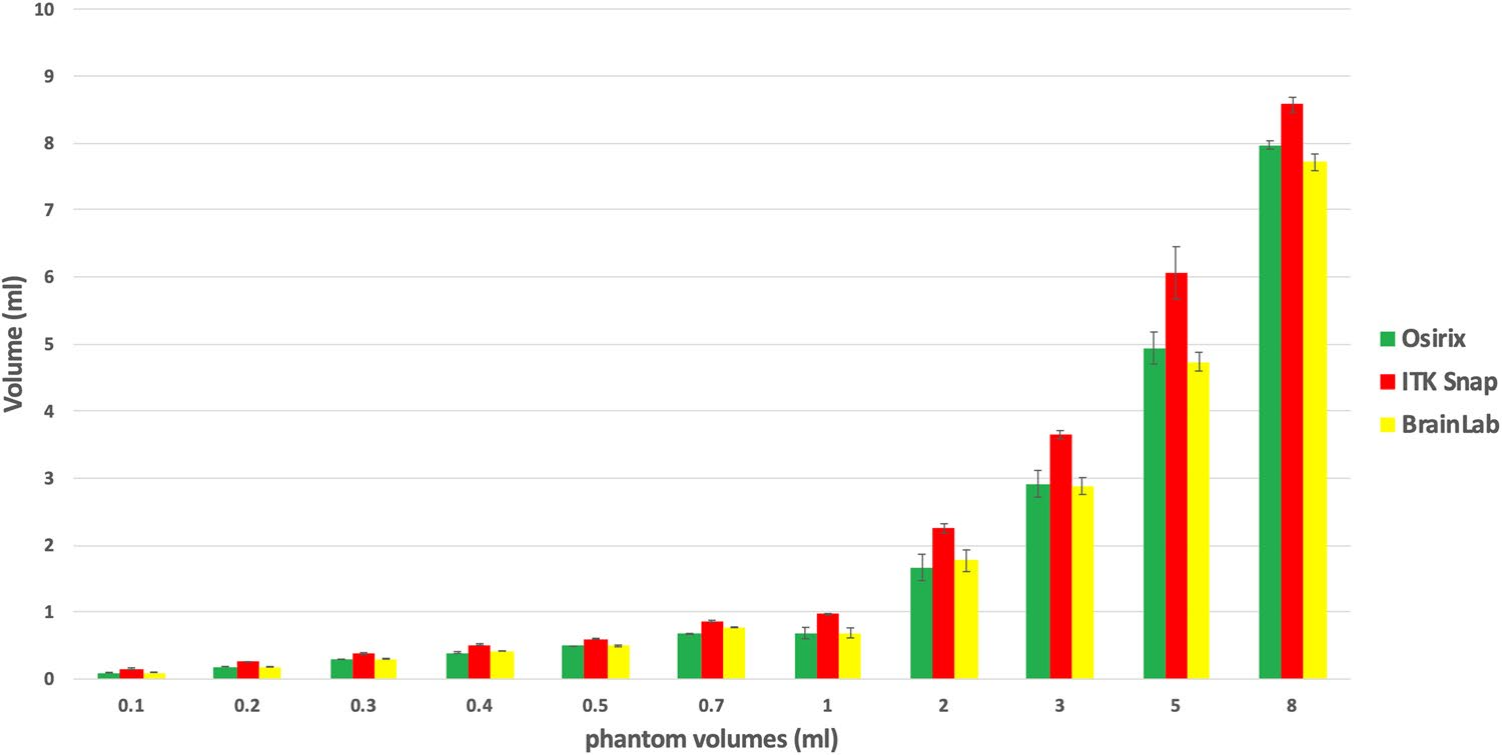
Comparison of phantoms. Volumetric results (bars) with indicated SD for microphantoms of 0.1 to 0.7 ml and phantoms of 1 to 8 ml (x-axis). The y-axis shows the calculated volume (mean) from the respective software tool.

Phantoms of six different known volumes below 1 ml (microphantoms) and another five different volumes from 1 to 8 ml were measured in triplicate using three semiautomated volumetric programs: OsiriX, ITK-Snap and BrainLab (Fig. 1). The volume estimates of all three software programs differed significantly from the actual volumes of the phantoms. The OsiriX and BrainLab software underestimated the size of the phantoms, on average, and ITK-Snap substantially overestimated the size of the phantoms.

The analysis of variance shows that the difference between the volumes estimated by the various softwares and the actual volume of the phantoms is significantly different (p < 10^–5^), but the residuals are not normally distributed, violating a key assumption of the analysis of variance. However, the Kruskal-Wallace test, which is non-parametric, confirmed that the overall difference between the volumes estimated by the various softwares and the actual volume of the phantoms is highly significantly different (p < 10^–10^).

Post-hoc Tukey analysis and pairwise t-tests were performed to compare the differences between the actual volumes of the phantoms to the volumes estimated by each pair of software programs to each other to determine where the differences lay. Both tests show the same result: ITK-Snap differs greatly from OsiriX and BrainLab, but the OsiriX and BrainLab estimates do not differ significantly from each other.

In order to determine if the difference between the estimated volume and true volume (Δ-volume) was greater for larger volume phantoms than for smaller volume phantoms, we used a linear regression of the phantom volume (independent variable) on the Δ-volume (dependent variable). The absolute magnitude of the difference between the estimated volume and true volume (Δ-volume) was significantly greater for larger volume phantoms than for smaller volume phantoms with BrainLab, but for ITK-Snap, the association was in the opposite direction (larger Δ-volume with smaller phantoms). There was no significant association with OsiriX.

Linear regression was also used to assess possible associations between the coefficient of variation and the true volume of the phantoms. No association was found between the coefficient of variation and the phantom volumes for OsiriX or ITK-Snap. Larger coefficients of variation were seen with larger phantom volumes for BrainLab (p < 0.001).

### Tumors

**Figure 2:**
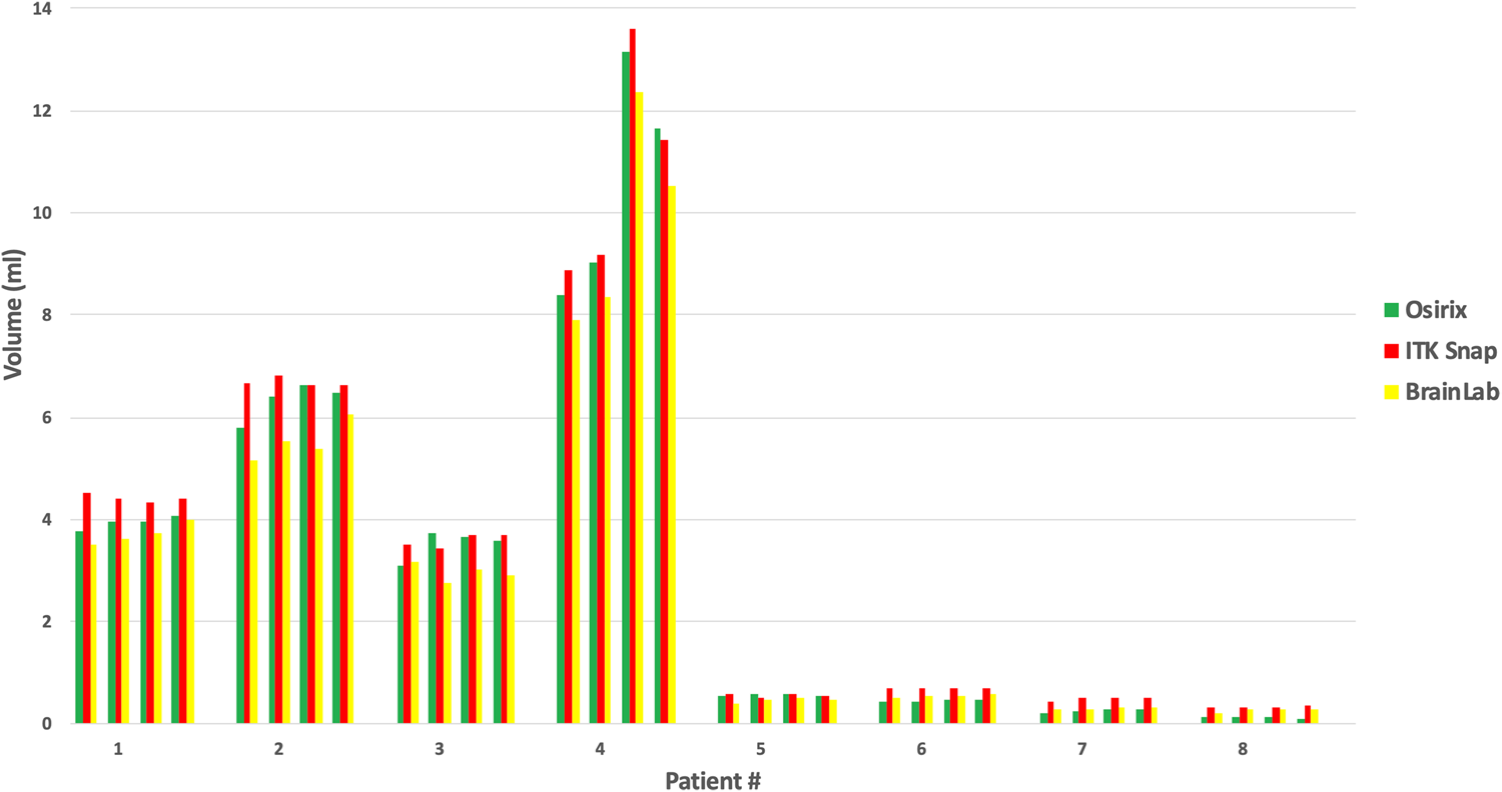
Comparison of tumors. Comparison of volumetric mean values in serial MRI scans of different tumors (patients 1 to 8).

The results of the VS volumetry are shown in Fig. 2. The true volumes of the VS tumors measured in the NF2 patients are unknown, but the precision of the semi-automated volume calculation of the software can be assessed by the coefficient of variation of the triplicate volume measurements for each of the five tumors on each exam (Table 2). In most cases, the coefficient of variation is less than 5%, which is considered to be good measurement reproducibility, for all three software programs.

**Table 2:** Patients: tumor volumes were estimated in triplicate in a total of 32 MRI scans with each of three different methods.

Parameter	Osirix	ITK-SNAP	BrainLab
**Patient 1**			
MRI #1			
Mean estimated volume (ml)	3.77	4.52	3.51
Standard deviation (ml)	0.09	0.13	0.02
Coefficient of variation	0.02	0.03	0.01
MRI #2			
Mean estimated volume (ml)	3.96	4.40	3.63
Standard deviation (ml)	0.02	0.06	0.02
Coefficient of variation	0.00	0.01	0.01
MRI #3			
Mean estimated volume (ml)	3.97	4.34	3.71
Standard deviation (ml)	0.04	0.12	0.13
Coefficient of variation	0.01	0.03	0.03
MRI #4			
Mean estimated volume (ml)	4.07	4.42	4.00
Standard deviation (ml)	0.04	0.07	0.13
Coefficient of Variation	0.01	0.02	0.03
**Patient 2**			
MRI #1			
Mean estimated volume (ml)	5.80	6.67	5.16
Standard deviation (ml)	0.04	0.03	0.10
Coefficient of variation	0.01	0.00	0.02
MRI #2			
Mean estimated volume (ml)	6.38	6.80	5.54
Standard deviation (ml)	0.07	0.12	0.02
Coefficient of variation	0.01	0.02	0.00
MRI #3			
Mean estimated volume (ml)	6.62	6.61	5.36
Standard deviation (ml)	0.08	0.20	0.01
Coefficient of Variation	0.01	0.03	0.00
MRI #4			
Mean estimated volume (ml)	6.46	6.61	6.46
Standard deviation (ml)	0.23	0.07	0.06
Coefficient of variation	0.04	0.01	0.01
**Patient 3**			
MRI #1			
Mean estimated volume (ml)	3.09	3.50	3.17
Standard deviation (ml)	0.07	0.13	0.04
Coefficient of variation	0.02	0.04	0.01
MRI #2			
Mean estimated volume (ml)	3.73	3.44	2.75
Standard deviation (ml)	0.06	0.07	0.06
Coefficient of variation	0.02	0.02	0.02
MRI #3			
Mean estimated volume (ml)	3.64	3.68	3.03
Standard deviation (ml)	0.11	0.13	0.06
Coefficient of variation	0.03	0.03	0.02
MRI #4			
Mean estimated volume (ml)	3.58	3.69	2.90
Standard deviation (ml)	0.02	0.02	0.02
Coefficient of variation	0.01	0.00	0.01
**Patient 4**			
MRI #1			
Mean estimated volume (ml)	8.39	8.86	7.90
Standard deviation (ml)	0.06	0.24	0.02
Coefficient of variation	0.01	0.03	0.00
MRI #2			
Mean estimated volume (ml)	9.03	9.18	8.35
Standard deviation (ml)	0.11	0.03	0.06
Coefficient of variation	0.012	0.00	0.01
MRI #3			
Mean estimated volume (ml)	13.16	13.61	12.35
Standard deviation (ml)	0.14	0.32	0.18
Coefficient of variation	0.011	0.023	0.015
MRI #4			
Mean estimated volume (ml)	11.64	11.42	10.52
Standard deviation (ml)	0.12	0.13	0.32
Coefficient of variation	0.01	0.01	0.03
**Patient 5**			
MRI #1			
Mean estimated volume (ml)	0.53	0.57	0.40
Standard Deviation (ml)	0.00	0.02	0.01
Coefficient of Variation	0.00	0.03	0.03
MRI #2			
Mean estimated volume (ml)	0.57	0.50	0.49
Standard deviation (ml)	0.01	0.02	0.01
Coefficient of variation	0.01	0.04	0.02
MRI #3			
Mean estimated volume (ml)	0.57	0.58	0.50
Standard deviation (ml)	0.01	0.03	0.01
Coefficient of variation	0.01	0.05	0.02
MRI #4			
Mean estimated volume (ml)	0.55	0.54	0.47
Standard deviation (ml)	0.01	0.05	0.01
Coefficient of variation	0.01	0.09	0.02
**Patient 6**			
MRI #1			
Mean estimated volume (ml)	0.42	0.68	0.49
Standard deviation (ml)	0.01	0.04	0.02
Coefficient of variation	0.02	0.06	0.05
MRI #2			
Mean estimated volume (ml)	0.44	0.70	0.53
Standard deviation (ml)	0.00	0.01	0.03
Coefficient of variation	0.01	0.02	0.05
MRI #3			
Mean estimated volume (ml)	0.46	0.68	0.56
Standard deviation (ml)	0.02	0.03	0.04
Coefficient of variation	0.03	0.04	0.08
MRI #4			
Mean estimated volume (ml)	0.48	0.69	0.60
Standard deviation (ml)	0.01	0.02	0.02
Coefficient of variation	0.02	0.03	0.03
**Patient 7**			
MRI #1			
Mean estimated volume (ml)	0.22	0.45	0.28
Standard deviation (ml)	0.01	0.03	0.01
Coefficient of variation	0.03	0.06	0.05
MRI #2			
Mean estimated volume (ml)	0.24	0.50	0.27
Standard deviation (ml)	0.01	0.00	0.01
Coefficient of variation	0.04	0.00	0.04
MRI #3			
Mean estimated volume (ml)	0.27	0.50	0.30
Standard deviation (ml)	0.02	0.04	0.01
Coefficient of Variation	0.09	0.07	0.01
MRI #4			
Mean estimated volume (ml)	0.28	0.49	0.32
Standard deviation (ml)	0.01	0.02	0.00
Coefficient of variation	0.02	0.04	0.02
**Patient 8**			
MRI #1			
Mean estimated volume (mll)	0.14	0.31	0.22
Standard deviation (ml)	0.00	0.01	0.02
Coefficient of variation	0.03	0.04	0.08
MRI #2			
Mean estimated volume (ml)	0.13	0.30	0.27
Standard deviation (ml)	0.01	0.01	0.02
Coefficient of variation	0.09	0.03	0.08
MRI #3			
Mean estimated volume (ml)	0.13	0.33	0.27
Standard deviation (ml)	0.01	0.01	0.02
Coefficient of variation	0.04	0.02	0.06
MRI #4			
Mean estimated volume (ml)	0.10	0.34	0.28
Standard deviation (ml)	0.01	0.02	0.02
Coefficient of variation	0.08	0.06	0.08

We calculated the means, standard deviations (SD) and coefficients of variation (CV) of the three volume estimates at each time point for each patient using each method.

We used repeated measures analysis of variance for the comparisons of patient tumors. The three software programs produce highly significantly different estimates of the volumes (p < 10^–5^) after sphericity correction by either the Greenhouse–Geisser (GG) or Huynh–Feldt (HF) method.

We performed post hoc analysis to determine which programs differed from the others and whether the exam number had any effect. We did this with pair-wise t-tests, adjusting for multiple comparisons: The tumor volumes estimated for each software differed significantly from those estimated by each of the others at each exam time.

### Measurement speed

**Figure 3:**
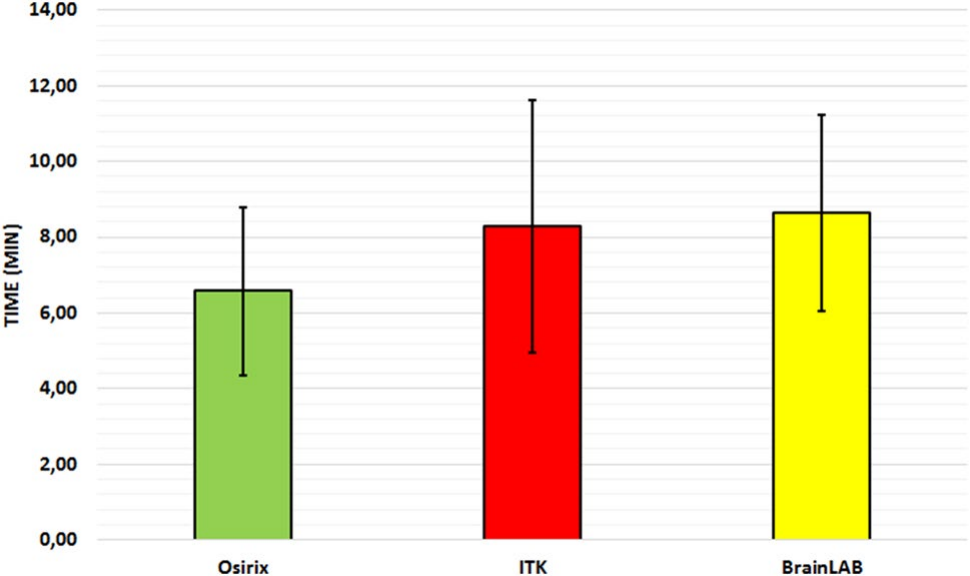
Comparison of time required for volumetric analysis. The difference between Osirx and ITK Snap (p < 0.0003) and Osirix and BrainLAB (p < 0.0001) is highly significant (patients 1 to 5).

Anova shows that the mean times required for performing volumetric analysis with these three software programs are significantly different (Fig. 3). Post-hoc Tukey HSD tests show that OsiriX is much faster than either of the other two programs. However, the time measurement is not strictly comparable as BrainLab runs on a different operating system and different hardware.

## Discussion

**Figure 4:**
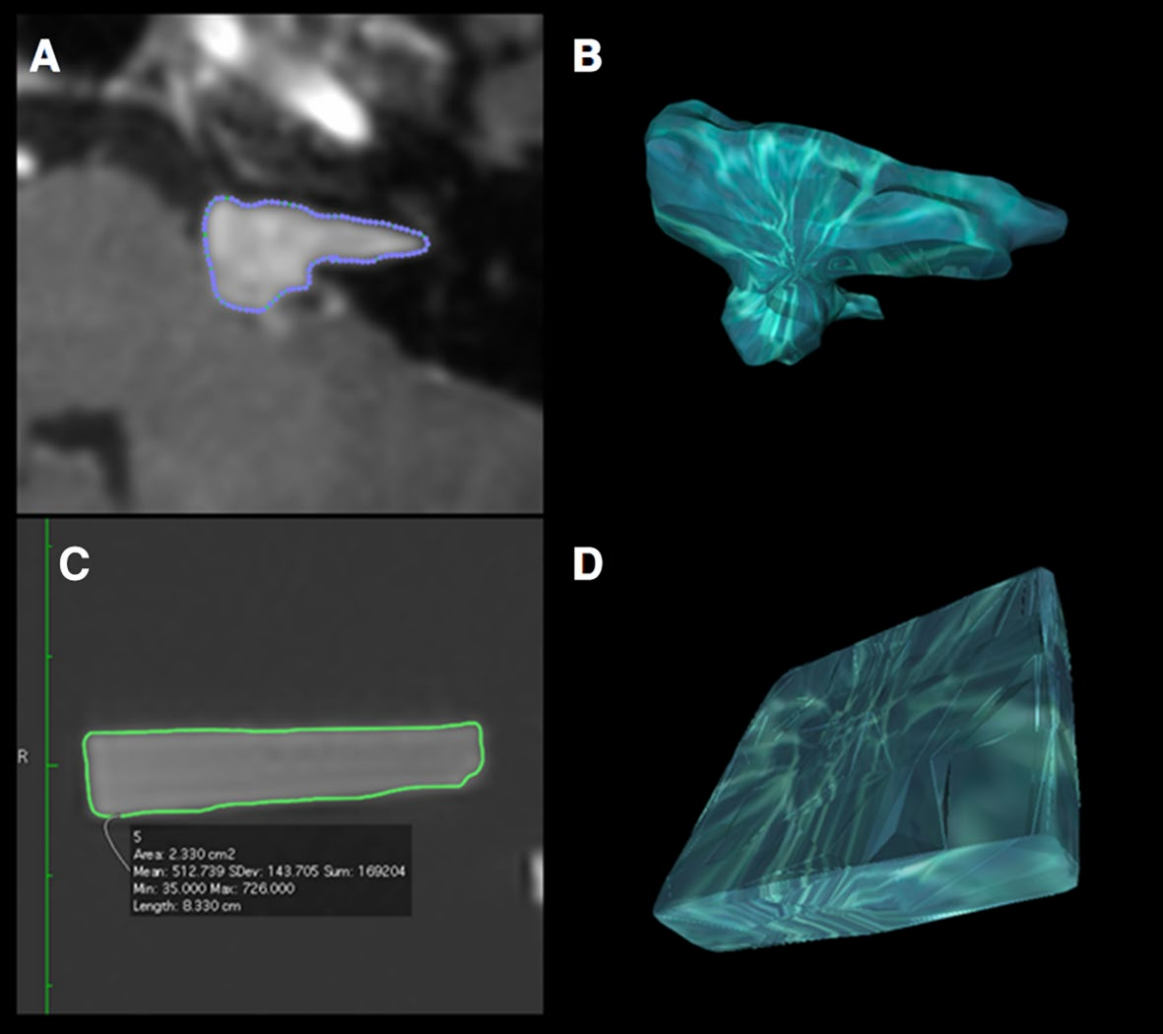
Typical display of volumetric measurement with OsiriX. (**A**) Semiautomated labeling of the tumor contouring in a VS. (**B**) Reconstructed 3D model of the VS. (**C**) Semiautomated registration of the phantom contours. D 3D model of the phantom.

We compared three different volumetric tools using calibrated phantoms and serial MRIs of patients with neurofibromatosis 2 and VS. We expected very accurate volume estimates for all of the phantoms because of their bright contrast and smooth, flat edges. However, none of the three software tools tested was able to calculate an accurate volume for the phantoms. In fact, OsiriX and BrainLab underestimated the actual size of the phantoms while ITK-Snap calculated volumes significantly above the real volume. The demarcation of the boundary is essential for calculating reliable volumes; this may be impeded in very small or insufficiently confined objects (Fig. 4). Both OsiriX and BrainLab provided volume estimates that were close to the true volumes of the 2 ml, 3 ml, 5 ml and 8 ml phantoms, but the volume estimates obtained with ITK-Snap were significantly greater than the true volumes of all of these phantoms. However, BrainLab produced even more accurate results for smaller phantom sizes than for larger. This association is remarkable, because it contrasts to the intuitive assumption that the precision of smaller measurements would be worse than for larger measurements. The relative miscalculation of the 1 ml phantom could be due to a blurrier border of the sharp edges (Figs. 1 and 4). Another factor might be the number of slices that cover one object. For flat objects the inter-slice volume-loss might be relatively bigger than for compact or spherical objects.

By comparing the sofware’s results to each other the volumes calculated by BrainLab and OsiriX seem to be at least comparable as the differences were not significant, while ITK-Snap produced significantly different results.

The patient tumor data set differs from phantom data set in two ways: (1) We do not know the true volume of the VS, so we cannot judge accuracy by comparison to the ground truth; we can only compare the measurements obtained by the software programs to each other; and (2) We have measurements of the tumors at four different times; the phantoms were just measured at one time.

Vestibular schwannomas in patients with NF2 usually progress over time^18^. Even in patients in whom bevacizumab treatment produces tumor regression, sustained progression usually resumes if treatment is discontinued^6^ and may be seen even if bevacizumab therapy is maintained^8^. Accurate and reliable volumetric measurement tools are, therefore, crucial for risk stratification and therapy. However, neither the absolute nor baseline volume of VS are crucial for the assessment of the natural history or therapy success; rather, variables like time to progression or growth rate are important outcome measures. The shape of the tumor as well as the distribution of the contrast agent may have an important influence on the accuracy and reproducibility of the calculated volume. Image quality, slice thickness and radiological experience are further factors influencing the result of volumetric analysis. Nevertheless volumetric analysis outmatches two-dimensional analysis by far^11,12,15,16^ and makes therapeutic decisions more reliable. To date many different software tools with more or less different algorithms exist that offer volumetric analysis. We aimed to test, whether important outcome measures differ using different volumetric tools. The three software tools studied here were all found to exhibit mostly precise estimates of VS volumes in NF2 patients independent from tumor size, but the volumes estimated by each of the softwares differed significantly for each timepoint. This systematic difference indicates that comparison of tumor volumes estimated on different platforms may be misleading. Estimates of the phantoms’ standard volumes of 2 ml or more made by OsiriX or BrainLab were more accurate than those made by ITK-SNAP, but we could not determine which software was more accurate in estimating tumor volumes in patients because the actual volumes of these tumors is not known.

The post-hoc Tukey HSD Test and paired t-tests show that the phantom volumes estimated by ITK SNAP were bigger than those estimated by BrainLab or OsiriX, but the phantom volumes estimated by those two programs did not differ from each other. In the case of the tumours, the volumes estimated by ITK SNAP were significantly greater than those estimated by OsiriX, which were in turn greater than those estimated by BrainLab. This systematic difference is important as it illustrates that volumetric values estimated with different software platforms cannot be compared reliably and that different mathematical algorithms may produce different results in regard to image quality, and tumor size. Also contrast intensity and shape of the target volume may play an important role.

Regarding the operating time, OsiriX seems to be much faster than ITK-Snap or BrainLab (post-hoc Tukey HSD Test). Tumor volume calculation is 1–4 min per analysis faster with OsiriX. As the BrainLab analysis is bound to the neurosurgical work station the comparison to the other two tools is not valid, because the processor-speed and operating system are different.

## Conclusion

Volumetric assessment of MRI images is associated to an intrinsic risk of error for all three tested volumetric tools. Moreover, different software tools exhibited systematic differences, meaning that volumetric measurements made using different software cannot be reliably compared.

For precise volumes and comparable results, it is mandatory to use the same volumetric tool for all measurements. However, all tools tested produce results that are clinically useful.

## Supplementary information

Supplementary information

## Data Availability

The datasets generated during and/or analyzed during the current study are available from the corresponding author on reasonable request.
