# Supplementary material for: MRI based volumetric measurements of vestibular schwannomas in patients with neurofibromatosis type 2: comparison of three different software tools
Source: Sci Rep. 2020 Jul 14;10:11541. doi: 10.1038/s41598-020-68489-y (PMC7360562; doi:10.1038/s41598-020-68489-y)
Supplement: Supplementary file 1 — Supplementary information [file 41598_2020_68489_MOESM1_ESM.pdf]

MRI based Volumetric Measurements of Vestibular Schwannomas in Patients with Neurofibromatosis Type 2: Comparison of Three Different Software Tools

Philipp Kollmann[1], Victor-Felix Mautner[1], Johannes Koeppen[2], Ralph Wenzel[3], Jan M. Friedman[4], Johannes Salamon[5] and Said Farschtschi[1]

[1] University Medical Center Hamburg-Eppendorf, Dept. Of Neurology, Hamburg, Germany
[2] University Medical Center Hamburg-Eppendorf, Dept. Of Neurosurgery, Hamburg, Germany
[3] Radiological Practice Altona, Hamburg, Germany
[4] University of British Columbia, Dept of Medical Genetics, Vancouver, Canada
[5] University Medical Center Hamburg-Eppendorf, Department of Diagnostic and Interventional Radiology and Nuclear MedicineDept. Of Radiology, Hamburg, Germany

Supplementary tables

In the following section two supplementary tables to the above-named article are listed. The tables contain all results (i.e. volume calculation of tumors and phantoms); the collected data have been analyzed according to the protocol described in the methods section in the body of the main manuscript.

Table S1: Tumors. Volumes were calculated in triplicate with each of three different methods on images taken during four separate examinations about three months apart.

Patient	Software	Exam	mean	standard deviation	coefficient of variation
1	BrainLab	1	3.51	0.02	0.01
1	BrainLab	2	3.63	0.02	0.01
1	BrainLab	3	3.71	0.13	0.03
1	BrainLab	4	4.00	0.13	0.03
1	ITK.SNAP	1	4.52	0.13	0.03
1	ITK.SNAP	2	4.40	0.05	0.01
1	ITK.SNAP	3	4.34	0.12	0.03
1	ITK.SNAP	4	4.42	0.07	0.02
1	Osirix	1	3.77	0.09	0.02
1	Osirix	2	3.96	0.01	0.00
1	Osirix	3	3.98	0.04	0.01
1	Osirix	4	4.08	0.04	0.01
2	BrainLab	1	5.16	0.10	0.02
2	BrainLab	2	5.54	0.02	0.00
2	BrainLab	3	5.36	0.01	0.00
2	BrainLab	4	6.05	0.06	0.01
2	ITK.SNAP	1	6.67	0.04	0.01
2	ITK.SNAP	2	6.80	0.11	0.02
2	ITK.SNAP	3	6.61	0.20	0.03
2	ITK.SNAP	4	6.61	0.07	0.01
2	Osirix	1	5.80	0.05	0.01
2	Osirix	2	6.38	0.07	0.01
2	Osirix	3	6.62	0.07	0.01
2	Osirix	4	6.46	0.23	0.04

Patient	Software	Exam	mean	standard deviation	coefficient of variation
3	BrainLab	1	3.17	0.04	0.01
3	BrainLab	2	2.75	0.06	0.02
3	BrainLab	3	3.03	0.06	0.02
3	BrainLab	4	2.90	0.02	0.01
3	ITK.SNAP	1	3.50	0.13	0.04
3	ITK.SNAP	2	3.44	0.07	0.02
3	ITK.SNAP	3	3.68	0.13	0.03
3	ITK.SNAP	4	3.69	0.02	0.00
3	Osirix	1	3.09	0.07	0.02
3	Osirix	2	3.73	0.05	0.01
3	Osirix	3	3.64	0.11	0.03
3	Osirix	4	3.58	0.02	0.00
4	BrainLab	1	7.90	0.02	0.00
4	BrainLab	2	8.35	0.06	0.01
4	BrainLab	3	12.35	0.18	0.01
4	BrainLab	4	10.52	0.32	0.03
4	ITK.SNAP	1	8.86	0.23	0.03
4	ITK.SNAP	2	9.18	0.03	0.00
4	ITK.SNAP	3	13.61	0.32	0.02
4	ITK.SNAP	4	11.42	0.13	0.01
4	Osirix	1	8.39	0.05	0.01
4	Osirix	2	9.03	0.11	0.01
4	Osirix	3	13.16	0.14	0.01
4	Osirix	4	11.64	0.12	0.01

Patient	Software	Exam	mean	standard deviation	coefficient of variation
5	BrainLab	1	0.400	0.010	0.025
5	BrainLab	2	0.487	0.012	0.024
5	BrainLab	3	0.500	0.010	0.020
5	BrainLab	4	0.470	0.010	0.021
5	ITK.SNAP	1	0.569	0.015	0.025
5	ITK.SNAP	2	0.502	0.019	0.037
5	ITK.SNAP	3	0.580	0.029	0.050
5	ITK.SNAP	4	0.536	0.045	0.084
5	Osirix	1	0.529	0.002	0.004
5	Osirix	2	0.567	0.003	0.006
5	Osirix	3	0.575	0.007	0.012
5	Osirix	4	0.545	0.006	0.011
6	BrainLab	1	0.494	0.025	0.050
6	BrainLab	2	0.532	0.026	0.049
6	BrainLab	3	0.559	0.043	0.077
6	BrainLab	4	0.599	0.015	0.026
6	ITK.SNAP	1	0.684	0.039	0.058
6	ITK.SNAP	2	0.697	0.013	0.019
6	ITK.SNAP	3	0.682	0.026	0.038
6	ITK.SNAP	4	0.689	0.023	0.033
6	Osirix	1	0.416	0.007	0.018
6	Osirix	2	0.442	0.004	0.010
6	Osirix	3	0.455	0.016	0.035
6	Osirix	4	0.482	0.010	0.020

Patient	Software	Exam	mean	standard deviation	coefficient of variation
7	BrainLab	1	0.281	0.014	0.048
7	BrainLab	2	0.272	0.010	0.038
7	BrainLab	3	0.300	0.002	0.007
7	BrainLab	4	0.323	0.005	0.015
7	ITK.SNAP	1	0.449	0.028	0.063
7	ITK.SNAP	2	0.498	0.002	0.005
7	ITK.SNAP	3	0.504	0.035	0.070
7	ITK.SNAP	4	0.494	0.017	0.035
7	Osirix	1	0.224	0.008	0.034
7	Osirix	2	0.237	0.009	0.040
7	Osirix	3	0.265	0.023	0.088
7	Osirix	4	0.276	0.007	0.025
8	BrainLab	1	0.222	0.018	0.082
8	BrainLab	2	0.267	0.022	0.081
8	BrainLab	3	0.268	0.017	0.063
8	BrainLab	4	0.285	0.023	0.081
8	ITK.SNAP	1	0.314	0.012	0.039
8	ITK.SNAP	2	0.305	0.011	0.035
8	ITK.SNAP	3	0.331	0.008	0.025
8	ITK.SNAP	4	0.343	0.019	0.055
8	Osirix	1	0.144	0.004	0.030
8	Osirix	2	0.127	0.011	0.087
8	Osirix	3	0.129	0.005	0.041
8	Osirix	4	0.101	0.009	0.085

Table S2: Phantoms. Volumes were calculated in triplicate with each of three different methods.

Phantom	Software	mean	standard deviation	coefficient of variation
0.1	BrainLab	0.102	0.004	0.035
0.1	ITK.SNAP	0.156	0.013	0.081
0.1	Osirix	0.097	0.003	0.033
0.2	BrainLab	0.183	0.004	0.023
0.2	ITK.SNAP	0.258	0.003	0.012
0.2	Osirix	0.188	0.001	0.006
0.3	BrainLab	0.303	0.007	0.022
0.3	ITK.SNAP	0.380	0.016	0.041
0.3	Osirix	0.296	0.002	0.007
0.4	BrainLab	0.420	0.003	0.007
0.4	ITK.SNAP	0.517	0.012	0.024
0.4	Osirix	0.397	0.014	0.035
0.5	BrainLab	0.495	0.013	0.026
0.5	ITK.SNAP	0.603	0.007	0.011
0.5	Osirix	0.490	0.001	0.001
0.7	BrainLab	0.774	0.006	0.008
0.7	ITK.SNAP	0.865	0.013	0.015
0.7	Osirix	0.674	0.008	0.011
1	BrainLab	0.69	0.08	0.11
1	ITK.SNAP	0.97	0.01	0.01
1	Osirix	0.69	0.09	0.12

Phantom	Software	mean	standard deviation	coefficient of variation
2	BrainLab	1.77	0.16	0.09
2	ITK.SNAP	2.25	0.07	0.03
2	Osirix	1.67	0.20	0.12
3	BrainLab	2.88	0.13	0.04
3	ITK.SNAP	3.65	0.06	0.02
3	Osirix	2.92	0.20	0.07
5	BrainLab	4.73	0.14	0.03
5	ITK.SNAP	6.06	0.39	0.06
5	Osirix	4.94	0.24	0.05
8	BrainLab	7.71	0.13	0.02
8	ITK.SNAP	8.57	0.11	0.01
8	Osirix	7.97	0.06	0.01
